# The role of long non-coding RNA ANRIL in the development of atherosclerosis

**DOI:** 10.1016/j.ncrna.2022.09.002

**Published:** 2022-09-06

**Authors:** Ilgiz Gareev, Valentin Kudriashov, Albert Sufianov, Sema Begliarzade, Tatiana Ilyasova, Yanchao Liang, Ozal Beylerli

**Affiliations:** a Educational and Scientific Institute of Neurosurgery, Рeoples’ Friendship University of Russia (RUDN University), 6 Miklukho-Maklaya St, Moscow, 117198, Russian Federation; b Gastric Cancer Center, West China Hospital of Sichuan University, China; c Department of Neurosurgery, Sechenov First Moscow State Medical University (Sechenov University), Moscow, Russia; d Republican Clinical Perinatal Center, Republic of Bashkortostan, 450106, Russia; e Department of Internal Diseases, Bashkir State Medical University, Republic of Bashkortostan, Ufa, 450008, Russia; f Department of Neurosurgery, The First Affiliated Hospital of Harbin Medical University, Harbin, 150001, China

**Keywords:** Long non-coding RNA, ANRIL, Atherosclerosis, Apoptosis, Cell proliferation

## Abstract

Atherosclerosis is an important pathological basis of coronary heart disease, and the antisense non-coding RNA in the INK4 locus (ANRIL) is located in the genetically susceptible segment with the strongest correlation with it - the short arm 2 region 1 of chromosome 9 (Chr9p21). ANRIL can produce linear, circular and other transcripts through different transcriptional splicing methods, which can regulate the proliferation and apoptosis of related cells and closely related to the development of atherosclerotic plaques. Linear ANRIL can regulate proliferation of vascular smooth muscle cells (VSMCs) in plaques by chromatin modification, as well as affecting on proliferation and the apoptosis of macrophages at the transcriptional level; circular ANRIL can affect on proliferation and apoptosis of VSMCs by chromatin modification as well as interfering with rRNA maturation. In this review we describe the evolutionary characteristics of ANRIL, the formation and structure of transcripts, and the mechanism by which each transcript regulates the proliferation and apoptosis of vascular cells and then participates in atherosclerosis.

## 1 Introduction

Coronary heart disease is an important cause of death in the world, and its important pathological basis is atherosclerosis. In the early stage of atherosclerosis, long-term mechanical change in blood flow, high-fat and high-glucose environment, inflammatory infection damage, etc. The pathological factors of injury and pro-apoptotic factors increase, leading to the increase of endothelial cell injury and apoptosis, and the destruction of vascular endothelial integrity [1]. Subsequently, macrophages are activated to recognize and phagocytose oxidized lipoproteins accumulated under the endothelium through their surface receptors to form foam cells, which constitute an important core of necrotic lipids [2]. Activated macrophages can also produce a large number of interstitial collagen fibers to participate in the construction of plaque fibrous caps, and maintain plaque stability by removing apoptotic cells. Insufficient macrophage proliferation and increased apoptosis can lead to insufficient plaque fibrous cap strength and easy rupture, and apoptotic cells that are not cleared in time can further activate thrombin, induce intraplaque thrombosis, and then lead to acute coronary events [3]. Activated endothelial cells and macrophages can promote the transition of adjacent vascular smooth muscle cells from a quiescent and tiling telescopic differentiation state to a dedifferentiated state by paracrine various growth factors, and migrate to the vascular intima under the action of various chemokines. Smooth muscle cells can secrete a large number of extracellular matrix components such as collagen and polysaccharide molecules [4], which further accelerates the accumulation of plaques.

Non-coding RNA (ncRNA) refers to RNAs that are not translated into proteins, mainly including microRNA (miRNAs), long non-coding RNA (lncRNA), and circular RNA (circRNA) [5,6]. They are transcribed from the genome but not translated into proteins, and perform their respective biological functions at the RNA level [7]. NcRNAs bind to many molecular targets to form regulatory networks, which in turn initiate specific cellular biological responses, with the function of regulating gene expression, influencing intracellular signaling, participating in epigenetic modifications and other life activities, and thus playing a role in the occurrence and development of tumors and other diseases [8,9].

LncRNAs are a class of RNAs with transcripts longer than 200bp that do not encode proteins [[10], [11], [12], [13]]. LncRNAs were considered to be “transcriptional noise” with no biological function in the early days, and are by-products of RNA polymerase II transcription, which have no biological function [[14], [15], [16], [17]]. LncRNAs are characterized by their large number, variety and mode of action. At present, there is no uniform classification standard for lncRNAs [[18], [19], [20]]. According to the localization of lncRNAs in cells, they can be divided into cytoplasmic lncRNAs and cytosolic lncRNAs, some of which are located in both the nucleus and the cytoplasm [21,22]. LncRNAs may play different regulatory functions according to their different cellular localization (Table 1) [23,24]. In the cytoplasm, lncRNAs can act as competing endogenous RNAs (ceRNAs) to compete with miRNAs for binding and contribute to the release of target mRNAs.

**Table 1:** Role of lncRNAs in atherosclerosis.

LncRNA	Pathophysiological Effects	Indirect targets & Signaling pathway	Ref.
ANRIL	Slows cell cycle gene expression		[[25], [26], [27], [28], [29]]
MALAT1	Migratory behavior, increases AKT pathway behavior in endothelial cells	CXCR2 and AKT, AKT pathway	[[30], [31], [32], [33], [34], [35]]
LOC100129973	Suppress apoptosis of endothelial cells	API5 and BCL2L12	[36]
MEG3	Suppresses migration, proliferation and tube formation of endothelial cells	RhoB and PTEN	[37,38]
LEENE	Regulates eNOS expression and EC function		[39]
LISPR1	Angiogenesis, vascular stability and permeability	S1P signaling pathway	[40]
SMILR	VSMC proliferation	HAS2	[41]
LincRNA-p21	Reduces cell proliferation and increases apoptosis	p53 feed forward loop	[42]
SENCR	Promotes migration, proliferation and tube formation of endothelial cells	CCL5, CEACAM1, and CX3CL1 (migratory and angiogenic genes)	[43,44]
MYOSLID	Activates VSMC contractile phenotype	TGFβ/SMAD, MYOCD/SRF pathways	[45]
H19	Reduces autophagy, apoptosis and reactive oxygen species in endothelial cells	MAPK and NF-kβ pathways	[[46], [47], [48]]

**Fig. 1:**
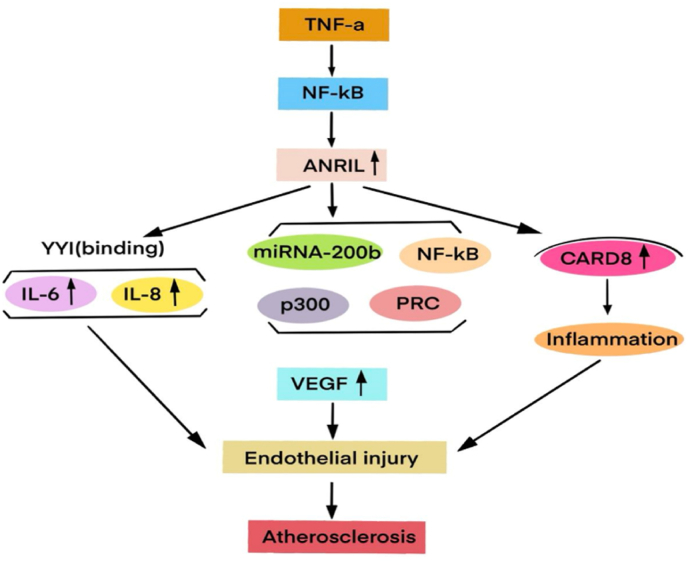
Role of lncRNA ANRIL in atherosclerosis.

The antisense non-coding RNA (antisense non-coding RNA in the INK4 locus, ANRIL) in the INK4 locus originated in placental mammals and acquired additional exons during evolution, and then part of the exons was gradually lost, and finally It is fully formed in apes [49]. ANRIL is located in the most susceptibility gene segment of human cardiovascular disease - the short arm 2 region 1 of chromosome 9 (Chr9p21), which can be distributed in the cytoplasm and nucleus [50]. Studies have shown that ANRIL is closely related to the occurrence of atherosclerosis [51,52]. Through the genome-wide association analysis study and subsequent studies of 15 596 patients with coronary heart disease and 34 992 control samples in Europe and Asia, it was found that Chr9p21 has the most cardiovascular disease-related single nucleotide polymorphism mutations [53]. It is the most genetically susceptible segment associated with coronary heart disease. Hu et all. conducted a meta-analysis on the correlation between ANRIL polymorphisms and coronary heart disease risk in different regions and ethnic groups in Asia, Europe, North America, etc., and found that a variety of mononucleotides such as rs1333040, rs1333049 and rs2383207 on ANRIL were more abundant [54]. There is a correlation between the state of the disease and the risk of coronary heart disease. By selectively preserving the transcriptional splicing process of different exons, ANRIL can combine the editing of precursor mRNA to generate a variety of transcripts, including linear ANRIL transcripts and circular ANRIL transcripts [55]. Coronary heart disease-related single nucleotide polymorphism risk mutation at Chr9p21 locus can regulate the alternative splicing of ANRIL and affect the expression level of different ANRIL transcripts [50]. Linear RNA is a class of non-coding linear transcripts longer than 200 nucleotides, which can be involved in endothelial cell dysfunction, inflammatory response, lipid and lipid in atherosclerosis by mediating cell signaling, chromatin modification, transcription and translation regulation, etc. [[56], [57], [58]]. Different from the traditional transcriptional splicing method of linear RNA, circular RNA can resist exonuclease digestion and degradation, and its structure is more stable [59]. Circular RNA can participate in a variety of cell proliferation, apoptosis and inflammatory signaling pathways by regulating the transcription and expression of target genes, acting as protein bridging molecules and other mechanisms, thereby affecting the occurrence and development of atherosclerosis [[60], [61], [62]]. Different ANRIL transcripts play an important role in promoting and protecting the occurrence and evolution of atherosclerosis (Fig. 1) [[50], [51], [52]].

This article reviews the research progress of ANRIL transcripts with different structures that affect the occurrence and development of atherosclerosis by regulating the proliferation and apoptosis of vascular cells.

## 2 ANRIL affects cell proliferation and apoptosis by interfering with ribosomal RNA maturation process

Last few years the function of circular ANRIL has also been gradually explored, and with the progression of atherosclerosis, the expression level of circular ANRIL in plaque lesion tissue gradually decreases [63,64]. Proteomic studies have found that overexpression of cyclic ANRIL can significantly increase the expression levels of 32 proteins, among which the nucleolar protein (pescadillo), which plays an important role in the processing of ribosomal RNA precursors and ribosome assembly, is closely related to the expression of cyclic ANRIL [65]. Cyclic ANRIL weakens the binding of ribosomal RNA precursors to exonuclease through competitive binding to nucleolar proteins, which affects the maturation of ribosomes, resulting in decreased smooth muscle cell proliferation and increased apoptosis [66]. Immunofluorescence staining showed that overexpression of cyclic ANRIL in smooth muscle cells can reduce a large number of nucleoli, nuclear damage, and increase apoptosis in smooth muscle cells, confirming that cyclic ANRIL can affect the process of ribosomal RNA maturation and processing by interfering with it [67]. Proliferation and apoptosis of smooth muscle cells can slow down the progression of atherosclerosis by removing excessively proliferating smooth muscle cells from plaques [68].

## 3 ANRIL affects cell proliferation and apoptosis through other mechanisms

Cyclic ANRIL can also be used as a molecular scaffold for chromatin modification complexes, affecting target gene expression by regulating the covalent modification of histones, thereby affecting cell proliferation and apoptosis; or as a dynamic scaffold, binding, storing or transport transcription factors to specific subcellular locations such as mitochondria, affecting cell proliferation and metabolism [69,70]. There are many miRNA complementary binding sites on some circular RNAs, which can capture miRNA and produce a “sponge effect”, reducing its negative gene regulation effect on target mRNA [71]. Whether cyclic ANRIL also has such an effective miRNA binding site remains to be further bioinformatic analysis. In addition, circular RNAs can also bind to effector proteins and affect their related signaling pathways [72]. Whether cyclic ANRIL can affect cell proliferation and apoptosis through the above mechanisms and participate in the occurrence and development of atherosclerosis is also worthy of our exploration and verification.

### 3.1 Anril affects cell proliferation through chromatin modification

Meseure et al. found by RNA co-immunoprecipitation method that linear ANRIL binds efficiently to polycomb family proteins such as CBX7 and SUZ12 [73]. Polycomb family proteins can regulate gene expression by initiating and maintaining epigenetic modifications of chromatin [74,75]. Knockout of linear ANRIL can disrupt the binding of polycomb family protein SUZ12 to Chr9p21 site, resulting in increased expression of cyclin-dependent kinase 2 inhibitor B (CDKN2B) gene at Chr9p21 site, and increased vascular smooth muscle expression. decreased cell proliferation [76]. Knockout of linear ANRIL can also reduce the methylation level of an important protein component of chromatin, that is, histone H3 methylation level, and the cyclin-dependent kinase 2 inhibitor A (CDKN2A) gene expression level is increased, which leads to a decrease in the level of vascular cell proliferation [67]. In addition, linear ANRIL can also mediate the binding of SUZ12 to the p15INK4b site, affecting the proliferation and metabolic activity of vascular smooth muscle cells [66]. All of these studies have shown that linear ANRIL can affect the proliferation of vascular smooth muscle cells through chromatin modification, thereby accelerating plaque accumulation in the late stage of atherosclerosis.

### 3.2 Anril affects cell proliferation and apoptosis by interfering with transcription

Linear ANRIL can affect the transcriptional expression levels of multiple genes and increase the risk of atherosclerosis by interfering with the transcription of target genes. Overexpression of linear ANRIL in macrophage cell lines can induce AEBP2, EZH2, Jumonji/Jmj Jarid2 C-domain family protein, MEL18 DNA-binding protein, YY1 transcriptional repressor proteins, Gli-Kruppel family proteins with transcription regulation functions, such as COREST/REST, and these proteins are enriched in Alu repeats with the most active transcription of genes in the chromosomal segment [77,78]. Alu repeats are important regulators of *trans*-action, which can activate gene transcription by binding to target gene promoters such as cyclins, and affect the level of target gene transcription and expression. Cytologically, it is manifested as increased macrophage proliferation, enhanced metabolic activity, and decreased apoptosis [79]. Holdt et al. overexpressed linear ANRIL after 25%, 33%, and 100% mutational disruption of the Alu transcription regulator sequence and found that overexpressed ANRIL enhanced macrophage proliferation and inhibited apoptosis with Alu degradation [75]. Also, linear ANRIL affects transcriptional expression of target genes, such as cyclins, by regulating the level of transcription, thereby affecting the proliferation and apoptosis of macrophages. Studies have found that the expression of linear ANRIL in peripheral blood mononuclear cells of 2880 subjects is closely related to cell proliferation and apoptosis and the transcriptional expression level of target genes, and It is confirmed that linear ANRIL affects the proliferation and apoptosis of macrophages by interfering with transcription, thereby affecting the stability of atheromatous plaques [80].

## 4 Conclusions

More and more evidences show that ANRIL plays an important role in atherosclerotic cardiovascular disease, but what mechanism affects cell proliferation and apoptosis and participates in the occurrence and development of atherosclerosis still needs further research. With the continuous emergence of new technologies, studies have discovered the existence of more ANRIL transcripts, and there are more means to detect the transcriptional expression levels of target genes and protein molecules in different pathways, which provides an important guarantee for more effective mechanism exploration. However, due to the presence of many common exon fragments among different ANRIL transcripts, it is difficult to distinguish it, and the expression level of ANRIL in peripheral blood is low, and the degradation rate of linear ANRIL in vitro is high. These factors can become a serious obstacle to the process of studying the effect of ANRIL on the mechanism of the onset and development of atherosclerosis. Therefore, it is necessary to create a more stable system and a more accurate measurement method to further study the mechanism of action of ANRIL.

## Author contributions

Ilgiz Gareev, Valentin Kudriashov and Albert Sufianov conceptualized and designed the study. All authors participated in the acquisition, analysis and interpretation of the data. Sema Begliarzade, Tatiana Ilyasova and Yanchao Liang drafted the manuscript. Ozal Beylerli contributed to critical revisions of the manuscript. All authors agreed on the journal to which the article would be submitted, gave final approval for the version to be published, and agreed to be accountable for all aspects of the work.

## Funding

None.

## Declaration of competing interest

The authors declare that no conflicts of interest exist.
